# Pharmacological Threat to Lungs: A Case Series and Literature Review

**DOI:** 10.7759/cureus.1232

**Published:** 2017-05-09

**Authors:** Omar Irfan, Jaleed A Gilani, Abeel Irshad, Babar Irfan, Javaid A Khan

**Affiliations:** 1 Medicine, The Aga Khan University; 2 Medicine, Dr Ziauddin Hospital; 3 Medicine, Jinnah Sindh Medical University (SMC); 4 Pulmonology, The Aga Khan University

**Keywords:** pneumonitis, interstitial lung disease, drug

## Abstract

Drug-induced organ damage stands as a prevalent yet much-neglected issue globally. Keeping in view it’s rising frequency, health care providers stand obliged to be well versed with the de-merits of the agents they prescribe. Drug therapies causing damage present with a non-specific clinical presentation, histological findings or radiology, which further elaborates on the necessity of a conscientious diagnosis. Pulmonary architecture ranging from the airways, lung parenchyma, mediastinum, pleura, pulmonary vasculature or the neuromuscular system, all can fall victim to the dreaded outcomes of this menace. In order to establish successful diagnosis, the definite temporal relation between initiation of drug therapy and the development of the respiratory symptoms needs to be drawn. The most common form of pharmacologically arising lung toxicity is drug-induced pneumonitis or interstitial lung disease. Unfortunately, there is no adequate data available to review the extensiveness of this medication-associated risk in Pakistan which further highlights the necessity of carefully monitoring this overlooked yet assessable malady. Furthermore, identification and surveillance of this drug attributed peril shall help diminish burden on healthcare resources of the country. We present three recent cases of different types of drug-induced lung damage under treatment at our University Hospital.

## Introduction and background

Drug-induced organ damage presents as a common dilemma globally. As per a Swedish study, adverse drug reactions stand as the seventh most common cause of death in Sweden alone [1] whereas, in the UK, they account for 0.9% of total hospital admissions [2]. Between 1999 and 2008, the number of adverse drug reactions worldwide increased annually by 76.8% and the in-hospital mortality rate increased by 10% [3]. To minimize the potential morbidity and mortality from the above-mentioned menace, it is required that all health care providers are well educated with respect to the possible life threatening effects of the medications they prescribe. This makes strict monitoring of drug therapy essential to ensure medicines remain health-friendly and don’t oppose the purpose they have been formulated for. Various etiologies jeopardize the entire purpose of drug prescription; namely the inability to recognize damage, wrong dosage and even prescription of the inappropriate medicine.

The lung is one of the vital organs of the human body and because of its large surface area falls victim to damage from various etiological agents, namely infectious diseases, allergens, neoplastic growth, traumatic injuries, pollutants and pharmacological agents. Lung damage due to drug therapy has a non-specific clinical presentation, histological findings, and radiology, thus setting strict demands for appropriate and prompt diagnosis. Around 400 medications are known to cause drug-induced respiratory diseases alone with the true frequency being unknown [4]. The response to a particular drug varies from person-to-person making the situation further challenging.

Drug-induced lung damage can involve the airways, lung parenchyma, mediastinum, pleura, pulmonary vasculature or the neuromuscular system. The most common form of pharmacologically arising lung toxicity is drug-induced pneumonitis or drug-induced interstitial lung disease (DILD). Though the literature covering such a perilous subject is, unfortunately, scarce, it points out that 10% of patients receiving chemotherapeutic agents develop fibrosis in their lungs [5]. Drug-induced pulmonary toxicity is underdiagnosed worldwide which makes the exact global incidence of interstitial lung disease (ILD) vague but approximately 2.5% to three percent of cases owe credit to this condition [6]. There is no such data available to review the prevalence of this pharmacological threat in Pakistan which further emphasizes the necessity of recognizing drug-induced lung damage in order to relieve the burden on healthcare resources of the country. We present three cases of different types of drug-induced lung damage under treatment at our University Hospital after exemption from The Ethics Review Committee (ID 4597). The objective is to highlight and create awareness about such damage stemming from this condition. Informed consent statement was obtained for this study.

## Review

### Case 1

**Figure 1:**
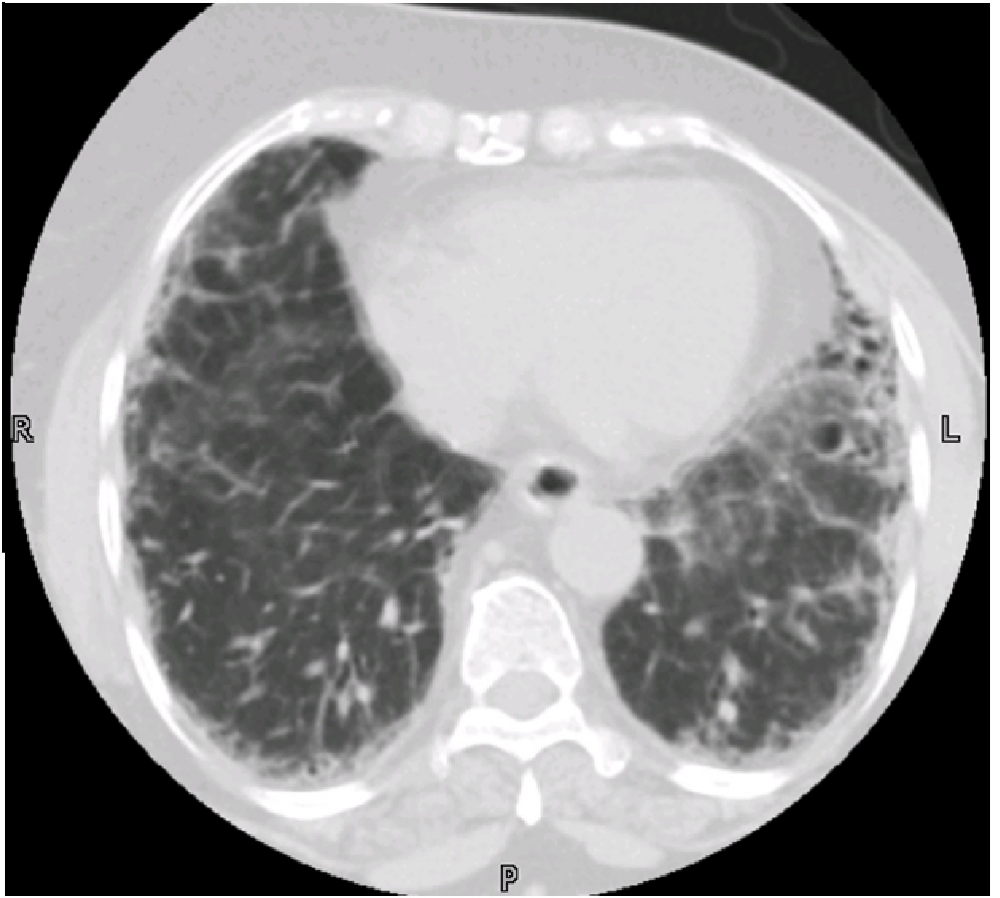
High-resolution computed tomography (HRCT) shows diffuse ground-glass opacities bilaterally

In June 2014, a 60-year-old postmenopausal female was presented to the outpatient clinic with a high-risk triple negative invasive ductal carcinoma of the breast. She began adjuvant treatment with Paclitaxel 80 mg/min, 126.4 mg IV over one hour, Doxorubicin 60 mg (97.2 mg IV over 15mins) and cyclophosphamide 600 mg/min (97.2mg IV over 30mins) every week. After the third and fourth cycles of chemotherapy, she developed progressive fatigue and a transient shortness of breath (SOB) lasting a few days. On auscultation, there were bilateral basal velcro type crackles with the peak flow of 400 ml pulmonary function test (PFT). The computed tomography (CT) scan of the chest showed diffuse ground-glass opacities bilaterally possible representative of hypersensitivity pneumonitis as shown in (Figure 1). A diagnosis of chemotherapy-induced hypersensitivity pneumonitis was made and further cycles of chemotherapy were canceled after weighing risk against benefit. Pulmonary function tests revealed reduced functional residual capacity and mild reduction in diffusing capacity. The patient was treated with prednisone at 1.5 mg orally daily. As both Paclitaxel and Cyclophosphamide can cause hypersensitivity pneumonitis [7], it was unclear which drug caused it. Doxorubicin was ruled out as it is not known to cause pulmonary toxicity [8]. Risk versus benefit of restarting the chemotherapy was weighed and it was decided to discontinue chemotherapy and keep the patient under surveillance. Over time, the SOB and cough improved but no significant change in the X-ray findings. The patient has a yearly mammogram and half yearly Pulmonology follow-up with Prednisone on once as a daily dose.

### Case 2

**Figure 2:**
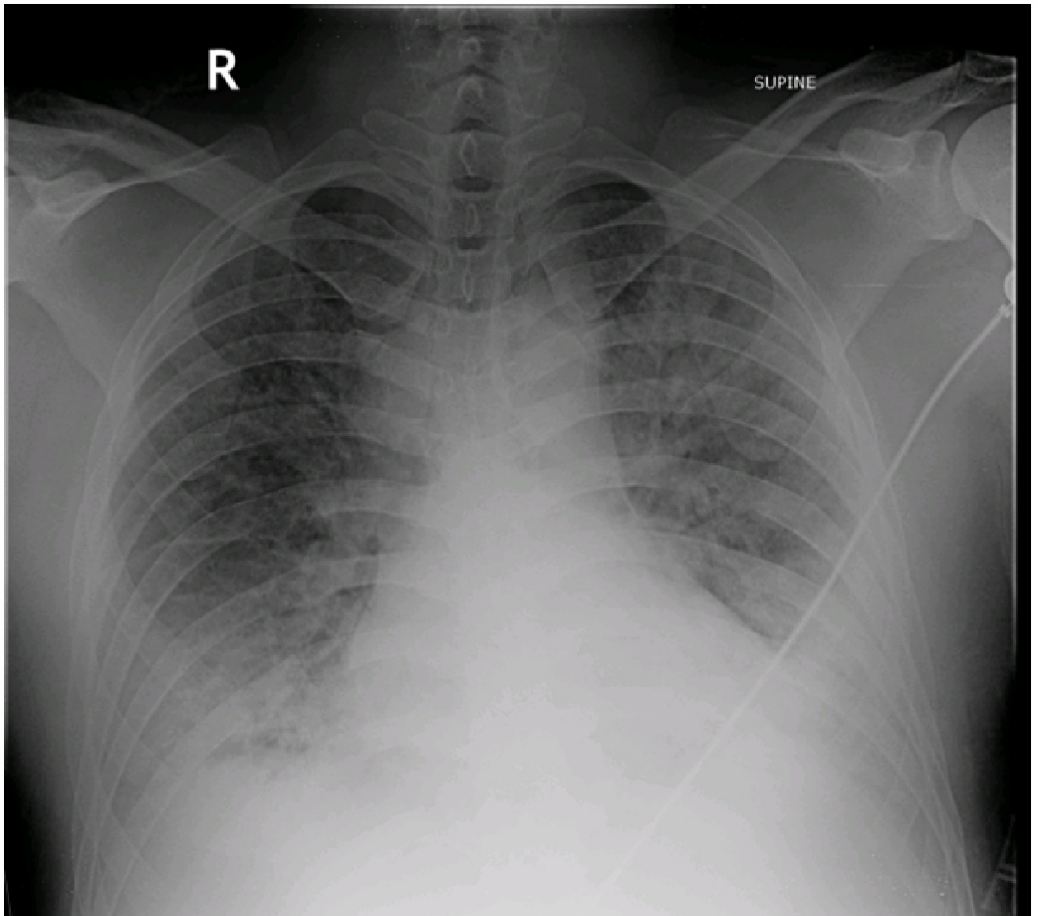
Chest Xray shows bilateral lower lobe infiltrates consistent with non-cardiac pulmonary edema

In July 2016, a 36-year-old male was presented in the emergency room (ER) with shortness of breath (SOB), dry cough and undocumented fever since one month. Two months earlier, the patient had been admitted for acute SOB and palpitations for a day. The patient was diagnosed with Atrial fibrillation on electrocardiogram (ECG). Amiodarone infusions were administered, along with a prescription for a 200 mg dosage of amiodarone to be taken every day. Four months later the patient presented to pulmonology clinic with SOB and cough for over a month. On physical examination, bilateral lower lung crepitations were heard. On further examinations, the chest X-radiation (X-ray) showed bilateral lower lobe infiltrates consistent with non-cardiac pulmonary edema as shown in (Figure 2). The high-resolution computed tomography (CT) scan revealed bilateral lower lobe shadowing, thickened septa and increased density in the thyroid and lung parenchyma. The patient was diagnosed with Amiodarone-induced interstitial pneumonitis leading to respiratory distress. He was intubated because of worsening hypoxia and remained on the ventilator for three days. He was discharged in stable condition on prednisone. The SOB and cough have now gradually subsided. The chest X-rays were done on follow-up visits which showed improvement over the subsequent weeks. The patient was started on Metoprolol because of his recent increasing heart rate.

### Case 3

**Figure 3:**
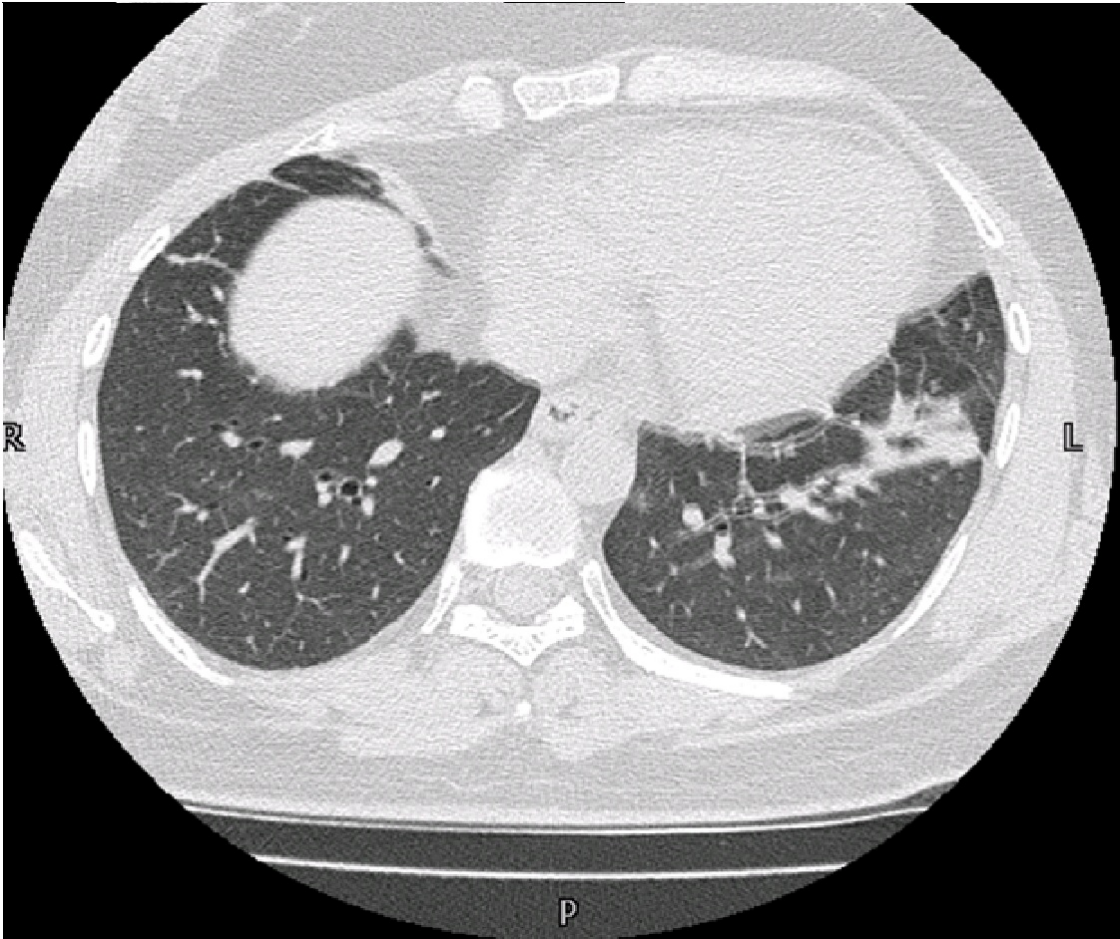
High-resolution computed tomography (HRCT) showing fibrosis and scarring more marked on the left than on right

A 36-year-old female, known case of Hodgkin's lymphoma presented on May 2016. She was started on Adriamycin, Bleomycin, Vinblastine, Dacarbazine (ABVD) therapy (Doxorubicin 44 mg, Bleomycin 15 mg, Vinblastine 10 mg, Dacarbazine 656 mg) for every two weeks. After the third cycle, the patient developed shortness of breath with the mild dry cough. She presented to the Pulmonology clinic with worsening shortness of breath over a month accompanied by a mild dry cough. On auscultation, there were end-inspiratory crackles and harsh vesicular breathing. Pulmonary function tests revealed reduced functional residual capacity and a reduction in diffusing capacity from 99% to 61%. The forced expiratory volume was decreased to around 50% with forced expiratory volume/forced vital capacity (FEV/FVC) of about more than 90% confirming a restrictive lung damage. The high-resolution computed tomography (HRCT) showed basal atelectasis and bilateral fibrosis as shown in (Figure 3). Bleomycin was stopped and the patient was started on oral steroids. She showed some improvement in her symptoms, but due to residual damage, the cough and dyspnea persisted even after few months of stopping her chemotherapy. On follow-up, the HRCT showed an increase in fibrotic bands bilaterally. On contrary, the respiratory symptoms have shown mild improvement.

### Discussion

The diagnosis of drug-induced lung disease (DILD) depends upon establishing a definite temporal relation between exposure to the causative agent and the development of the respiratory symptoms and signs. It is vital that all other causes of lung damage be excluded to ensure accurate diagnosis of this condition [9].

### Mechanisms

Various different mechanisms have been postulated to explain the mechanism of drug-induced lung damage. The first one is direct damage to the pneumocytes either via chemical injury to normal lung tissue [9] or in the case of bleomycin, the release of free reactive oxygen species [9-10]. Capillary leak syndrome, which is direct damage to the endothelium of the pulmonary vessels leading to increased vessel permeability and ultimately, pleural effusion, has been suggested as the second method [11]. While the third mechanism proposes an acute or delayed hypersensitivity reaction to explain the underlying pathophysiology [9, 11]. Drugs themselves can act as haptens or antigens that can lead to immune-mediated lung damage [9, 12]. Fibrogenesis following the inflammatory reaction has also been implicated in the development of drug-induced interstitial lung disease [13].

### Drugs

Paclitaxel, Doxorubicin, and cyclophosphamide are common adjuvants to breast cancer therapy [11]. Cyclophosphamide-induced lung toxicity is a rare adverse event (less than one percent), even if administered concurrently with radiotherapy or with other drugs [14]. Likewise, Paclitaxel-induced pulmonary toxicity is rare and unpredictable, with an incidence reportedly of being 0.7–12% [11]. In our case, one patient on all three drugs, Paclitaxel, Doxorubicin, and cyclophosphamide, developed lung disease as a side effect of therapy.

Amiodarone is a bi-iodinated derivative of benzofuran [15]. It is classified as a Class III anti-arrhythmic agent (Vaughan Williams Classification) [15-16]. In spite of reportedly having adverse effects (hyperthyroidism, corneal deposition, and skin photosensitization) [17-18] it has proven itself to be a reliable anti-arrhythmic drug [15]. However, the most potent side effect associated is amiodarone-induced pulmonary toxicity [18].

Four forms of pulmonary toxicity have been reported: Chronic Interstitial pneumonitis which is most common; organizing pneumonia with or without Bronchiolitis obliterans; acute respiratory distress syndrome (ARDS) though this occurs rarely and lastly, a solid pulmonary mass [16]. Amiodarone, being an amphiphilic cationic compound, damages type II pneumocytes by interfering with the movement of phospholipids across intracellular membranes [15, 19]. It also stops phospholipid catabolism via its inhibitory effect on lysosomal phospholipase [15, 19]. Likewise, amiodarone also causes an indirect immunologic reaction in genetically predisposed patients which results in lung parenchymal injury [20].

The incidence of Amiodarone-induced pulmonary toxicity varies from five percent to 15% and is correlated with dosage, the age of the patient, and preexisting lung disease [15-16]. Individuals receiving a dose of 400 mg or more daily for more than two months or a lower dose, commonly 200 mg daily for more than two years are at greatest risk for developing lung disease [19] which was also seen in our series. Toxicity is also reported more frequently in the male gender putting them at higher risk [19]. The mortality rate from amiodarone pulmonary toxicity has ranged widely in the literature but Papiris, et al. have reported it to be 10% for pneumonitis, 20%-30% for patients admitted to a hospital and 50% for those who develop ARDS based on their literature review [15].

Bleomycin associated lung damage, as seen in one of our cases, is the most frequently reported drug-induced lung disease with Bleomycin-induced pneumonitis (BIP) occurring in zero percent to 46% of the patients receiving the drug for chemotherapy [16, 21]. Bleomycin exerts its anti-tumor effect by forming free radicals leading to cell death but can cause pulmonary toxicity as a side effect [9]. The fact that the lungs are relatively deficient in the hydrolase enzyme responsible for detoxifying bleomycin results in its accumulation in the lung which further exacerbates this condition [9]. Bleomycin toxicity has been widely reported and studied in the local literature [22-23]. Toxicity can be fatal and in a retrospective study by Simpson, et al. patients aged over 40 with less than normal renal function had a greater than 10% risk of developing fatal toxicity [24]. Table 1 shows the common drugs with their possible lung toxic effects.

**Table 1:** Drugs with toxic pulmonary effects

Condition	Drug
Bronchiolitis obliterans organizing pneumonia	Amiodarone, Amphotericin, Bleomycin, Carbamazepine, Cocaine, Cyclophosphamide, Interferon alfa, Interferon beta, Methotrexate, Penicillamine, Phenytoin, Sulfasalazine, Tetracyclines
Interstitial pneumonia	Adalimumab, Amphotericin B, Amiodarone, Azathioprine, Bleomycin, Busulfan, Chlorambucil, Cyclophosphamide, Etanercept, Flecainide, Gold, Interferon alfa, Interferon beta, Infliximab, Melphalan, Methadone, Methotrexate, Mexiletine, Nitrofurantoin, Paclitaxel, Penicillamine, Phenytoin, Rituximab, Sirolimus, Statins, Sulfasalazine
Granulomatous pneumonitis	Cocaine, Cromolyn sodium, Fluoxetine, Methotrexate, Nitrofurantoin, Pentozocine, Procarbazine
Hypersensitivity pneumonitis	Azathioprine, 6-Mercaptopurine, beta-blockers, Busulfan, Fluoxetine, Nitrofurantoin, Procarbazine

### Diagnosis

As stated above, drug-induced lung disease is a diagnosis reached after excluding other causes, and an objective assessment of the patient’s baseline pulmonary status as well as treatment history is vital to differentiate drug-induced disease from a primary pathology. The diagnostic criteria set up by Irey, et al. to diagnose drug reactions is very helpful in assessing the patient [25] which is applicable in our cases as well.

The physical findings of drug-induced lung damage are non-specific and can reveal crackles and digital clubbing [9] while the time for onset varies from a few days to a few years. Workup should include a careful examination, laboratory studies, chest radiography and/or high-resolution CT. High-resolution CT is the currently the best non-invasive method to assess drug-induced lung disease [9]. The most common HRCT finding indicative of hypersensitivity pneumonitis is bilateral patchy ground-glass opacities with centrilobular ill-defined nodules in the upper lobe, as was also seen in one of our cases [12]. Lung consolidation and fibrosis can also be commonly seen [11].

### Management

There are no firm guidelines for the treatment of drug-associated interstitial lung disease and therapy tends to be on an empirical basis [26]. Withdrawal of the medication causing the disease is the first step in management followed by supportive care, which will result in the symptoms ultimately remitting [12]. For patients in respiratory distress, methylprednisolone (1 mg/kg/day or 60mg/day) is commonly used, again with gradual dose reduction [26]. In the case of oral corticosteroid contraindication, low-dose methylprednisolone (10–20 mg) is prescribed for patients with mild radiological or pulmonary function abnormalities [26]. This was what was done in our case series as well.

## Conclusions

In conclusion, drug-induced lung disease is an important but frequently overlooked differential in clinical practice. It is of cardinal importance to keep this differential in mind for patients on chemotherapy since they are very prone to develop this condition. Halting the offending drug, instituting supportive therapy and corticosteroids, regular follow-up in the clinic and radiological monitoring to assess the status of the patient’s condition is the mainstay in managing drug-induced lung disease. Through our case series, we aim to highlight and emphasize the importance of this disorder and hope those other institutions will share their experiences with this condition to further raise awareness about such a disease which is particularly prevalent but sadly overlooked in the developing world.
